# Two Oppositely Localised Frizzled RNAs as Axis Determinants in a Cnidarian Embryo

**DOI:** 10.1371/journal.pbio.0050070

**Published:** 2007-03-13

**Authors:** Tsuyoshi Momose, Evelyn Houliston

**Affiliations:** Université Pierre et Marie Curie (Paris VI), Centre National de la Recherche Scientifique Unité 7009 “Biologie du Développement,” Observatoire Océanologique, Villefranche-sur-Mer, France; University of California Berkeley, United States of America

## Abstract

In phylogenetically diverse animals, including the basally diverging cnidarians, “determinants” localised within the egg are responsible for directing development of the embryonic body plan. Many such determinants are known to regulate the Wnt signalling pathway, leading to regionalised stabilisation of the transcriptional coregulator β-catenin; however, the only strong molecular candidate for a Wnt-activating determinant identified to date is the ligand Wnt11 in *Xenopus.* We have identified embryonic “oral–aboral” axis determinants in the cnidarian Clytia hemisphaerica in the form of RNAs encoding two Frizzled family Wnt receptors, localised at opposite poles of the egg. Morpholino-mediated inhibition of translation showed that CheFz1, localised at the animal pole, activates the canonical Wnt pathway, promotes oral fates including gastrulation, and may also mediate global polarity in the ectoderm. CheFz3, whose RNA is localised at the egg vegetal cortex, was found to oppose CheFz1 function and to define an aboral territory. Active downregulation mechanisms maintained the reciprocal localisation domains of the two RNAs during early development. Importantly, ectopic expression of either CheFz1 or CheFz3 was able to redirect axis development. These findings identify Frizzled RNAs as axis determinants in *Clytia,* and have implications for the evolution of embryonic patterning mechanisms, notably that diverse Wnt pathway regulators have been adopted to initiate asymmetric Wnt pathway activation.

## Author Summary

How do different animal body parts form in the correct arrangement during development? Often, the explanation is provided by “determinant” molecules, prepositioned in the egg cell before it is fertilised. These determinant molecules initiate spatially localized programmes of gene expression, causing the various body parts to form in the appropriate place. Many determinants work by activating the Wnt signalling pathway; however, few concrete examples of determinant molecules have yet been discovered. We have found a new example of such a molecule by studying embryos of a jellyfish called *Clytia.* This molecule, found on one side of the egg, belongs to the “Frizzled” group of membrane proteins that activate Wnt signalling. Unexpectedly, we also found a second type of Frizzled molecule on the other side of the egg, which has a counterbalancing role in the embryo. Comparison of our findings in *Clytia* with those in other animals suggests that the molecular mechanisms responsible for body patterning via asymmetric Wnt pathway activation have not been tightly constrained during evolution.

## Abstract 3

The axis of a cnidarian is specified by the location of two maternal Frizzled mRNAs, revising the view that cnidarian axes are specified by the first cleavage initiation site.

## Introduction

The body plan of multicellular animals, generally defined in terms of axes of polarity, planes of symmetry, and germ layer organisation, emerges during early embryogenesis through a series of symmetry-breaking processes acting within and between cells [1]. The initial spatial cues that trigger these processes are frequently provided by maternal “determinants” localised at different sites within the egg. RNAs encoding Bicoid (transcription factor) and Nanos (RNA binding protein) localised at opposite poles of *Drosophila* eggs provide classic examples of determinants [2]. In many other species, unidentified determinants are deduced to act as regulators of the canonical Wnt signalling pathway. Activation of this pathway, generally by the binding of Wnt ligands to Frizzled transmembrane receptors, blocks constitutive degradation of the transcriptional coregulator β-catenin by a mechanism involving the cytoplasmic protein Dishevelled and inhibition of the kinase GSK3β [3,4]. Localised determinants cause β-catenin stabilisation in a restricted domain around the vegetal pole in early ascidian and sea urchin embryos, promoting endoderm/mesoderm fates, and in domains offset from the vegetal pole in amphibians and fish, promoting the establishment of a dorsal organiser centre [5–8]. A recent study revealed early regionalised stabilisation of β-catenin also in embryos of a cnidarian, the sea anemone *Nematostella,* indicating that it is an evolutionarily ancient component of early embryonic patterning [9].

The Cnidaria, which include sea anemones, corals, and jellyfish, provide an informative evolutionary perspective for the understanding of core developmental mechanisms because they form a sister group to the more complex three-layered and bilaterally symmetrical animals (protostomes and deuterostomes), and possess a very similar repertoire of regulatory genes [10–13]. Our laboratory model *Clytia (=Phialidium) hemisphaerica* exhibits the typical simplicity of cnidarian organisation in its hydra-like polyp form: two germ layers, called ectoderm and endoderm (or entoderm), and a single opening marking the “oral” end. The defining axis of oral–aboral polarity is first distinguishable at the onset of gastrulation, which proceeds by ingression of presumptive endoderm cells at the future oral pole of a hollow blastula [14]. Gastrulation produces simple polarised “planula” larvae from which polyps later form by metamorphosis. Experimental manipulations in another hydrozoan-group cnidarian, *Podocoryne carnea,* have shown that determinants responsible for the development of oral fates, including endoderm, and of global polarity properties such as directed swimming, are localised around the animal pole of the egg [15]. The animal pole of the *Clytia* egg correspondingly gives rise to the oral pole of the planula, although the strong regulative properties of the *Clytia* embryo make it harder to demonstrate the existence of maternal determinants [16,17]. Like the vegetally localised determinants in sea urchins, ascidians, amphibians, and fish, animally localised determinants in cnidarians are predicted to include canonical Wnt pathway activators [11,18], since β-catenin is stabilised in the future oral half of *Clytia* (this study) and *Nematostella* [9] embryos from as early as the 32-cell stage. Furthermore, Wnt pathway activation appears to promote formation of endoderm in *Nematostella* embryos and of the “posterior” (future oral) pole in *Hydractinia* [9,19], while in *Hydra* polyps it is associated with the oral/hypostome organiser region [18,20,21].

Molecular identification of the maternally localised determinants responsible for regional β**-**catenin stabilisation in early embryos remains an important goal for developmental biologists. Only in the amphibian *Xenopus* has a clear candidate been identified, the ligand Wnt11 [22]. The aim of this study was to identify localised maternal determinants responsible for regionalised β-catenin stabilisation in the oral half of the *Clytia* embryo. We focused on receptor or downstream Wnt pathway components rather than ligands, since in situ hybridisation analysis of *Nematostella* Wnt gene expression suggests that they play little or no maternal role; (ubiquitous) maternal RNA was originally detected for only one of the *Wnt* gene repertoire *(NvWnt11)* [23], and even this has now been put in doubt [18]. We were able to characterise both an animally localised Wnt pathway activating determinant, the receptor CheFz1, and a related vegetally localised determinant with opposing function, CheFz3.

## Results

### Two Clytia frizzled Genes Expressed in Early Embryos

**Figure 1:**
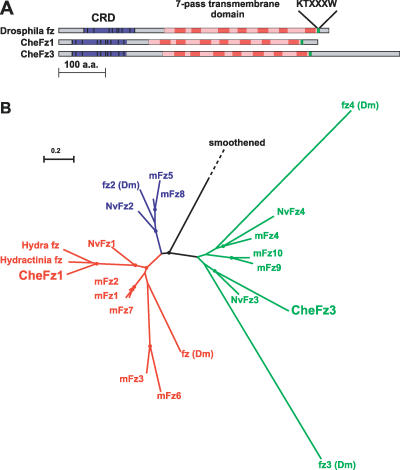
Two *Clytia* Frizzled Genes Belonging to Distinct Families (A) Domain structure of CheFz1 and CheFz3 proteins compared to the *Drosophila* Frizzled protein. Blue boxes: cysteine-rich domains; Pink/red boxes: seven-pass transmembrane domains (transmembrane regions in red); green boxes: KTXXXW motif. (B) Maximum likelihood analysis of relationships of Frizzled sequences from mouse (m), Drosophila melanogaster (Dm), and the known cnidarian Frizzleds: *Hydra vulgaris, Hydractinia echinata,* and N. vectensis (Nv). The tree was rooted using the *Drosophila* Smoothened sequence. Scale indicates number of inferred substitutions per site.

Two distinct *C. hemisphaerica* Frizzled sequences were identified by screening an embryo cDNA library and from searching an expressed sequence tag collection [24]. Both of the *Clytia* cDNAs identified encode classical Frizzled family receptors containing seven transmembrane segments, a cysteine-rich domain implicated in ligand binding, and a KTXXXW motif essential for the activation of the Wnt–β-catenin pathway [25,26] (Figure 1A). Comparison of their sequences with known Frizzled family genes grouped them with Drosophila fz (vertebrate *frizzled* 1/2/3/6/7) and *Drosophila fz3 and fz4* (vertebrate *frizzled 4, 9,* and*10*), and they were named *CheFz1* and *CheFz3* in line with the *Drosophila* genes (Figure 1B). The Nematostella vectensis genome trace archive also contains Frizzled family sequences in both these groups, as well as in the *Drosophila fz2* (vertebrate 5/8) group [18], indicating that the main *frizzled* gene subfamilies were founded before the bilaterian-cnidarian divergence.

### Opposite Localisation of Maternal CheFz1 and CheFz3 RNAs

**Figure 2:**
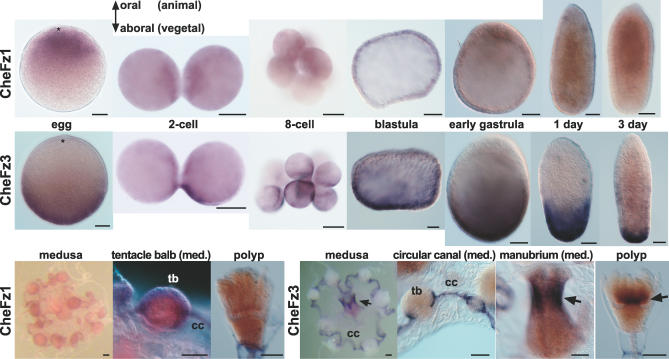
Opposing Localisation of CheFz1 and CheFz3 RNAs during Early Development In situ hybridisation of C. hemisphaerica eggs, embryos, and planula larvae (fixed at 1 and 3 d after fertilisation), showing the concentration of CheFz1 RNA (top row) in the animal cytoplasm of the egg and around the nuclei during first cleavage, and its graded oral–aboral distribution in blastula and early gastrula stages. CheFz1RNA levels declined during gastrulation and subsequently remained low throughout all regions, with some increase in the oral endoderm during planula development. CheFz3 RNA (middle row) was localised strongly to the vegetal egg cortex, becoming concentrated in the vegetal part of the cleavage furrow during cytokinesis, and then to the presumptive aboral pole throughout embryonic and larval development. Asterisks mark nuclei in eggs. In medusa and polyp stages (bottom row), low levels of CheFz1 RNA were detected in all regions, including the tentacle bulb (tb), while CheFz3 RNA was detected strongly in specific regions of the endoderm: the circular canal (cc) and a juxta-oral band of the manubrium (arrows). All embryos and polyps are oriented with the oral pole pointing up. Bars = 40 μm.

Whole mount in situ hybridization of unfertilised eggs revealed that both CheFz1 and CheFz3 RNAs are present as maternal transcripts and, remarkably, that they are distributed with complementary polarised localisations (Figure 2). Maternal CheFz1 RNA was found concentrated within the cytoplasm of the half of the egg containing the nucleus (i.e., the future oral half of the embryo). The predominantly oral localisation of CheFz1 RNA was maintained until the start of gastrulation. At the two- and four-cell stages it remained concentrated in cytoplasmic clouds close to the animally positioned nuclei, and at the eight- and 16-cell stages it became localised to roughly half the blastomeres. In blastulae and early gastrulae, CheFz1 RNA was detected as a gradient, the highest levels coinciding with the gastrulation initiation site. The level of RNA subsequently declined, the low signal detected in late gastrulae and young planula being stronger in the endodermal region. During planula development, CheFz1 RNA levels increased again in the oral endoderm, consistent with the expression of many Wnt genes in *Nematostella* at equivalent stages [23]. In, medusae and polyps, the low detectable levels of RNA were also concentrated in the endoderm, in line with the distributions of *frizzled-1* subfamily RNAs described in *Hydractina* and *Hydra* [27,28].

The distribution of CheFz3 RNA was entirely opposite to that of CheFz1 RNA during early development. In eggs, it was localised to a domain on the future aboral side of the embryo (i.e., opposite the egg nucleus), and confined to a thin cortical layer rather than to the cytoplasm. The aboral cortical domain of CheFz3 RNA was inherited by daughter blastomeres during cleavage divisions. CheFz3 RNA was later found to be strongly localised in the aboral half of blastulae and early gastrulae, opposite the gastrulation initiation site. In contrast to CheFz1 RNA, CheFz3RNA remained strongly detectable during gastrulation and larval development, becoming progressively more tightly restricted to the aboral pole of the planula. *CheFz3* was selectively expressed in two endodermal regions in medusae: the circular canal, and a band of the manubrium offset from the oral opening, as well as in an equivalent juxta-oral band of the manubrium in polyps.

### CheFz1 Is Required for β-Catenin Stabilisation and Oral Fate Specification

**Figure 3:**
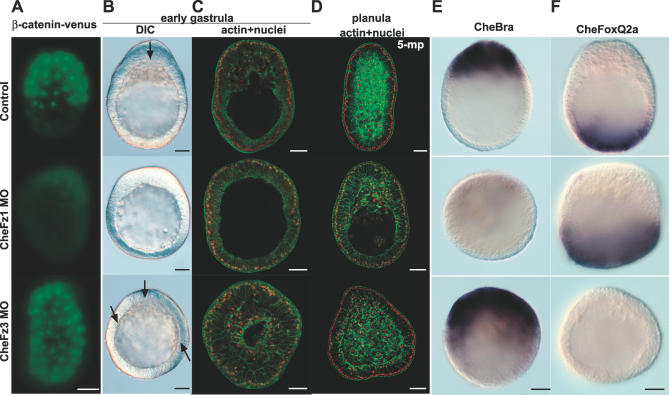
CheFz1 and CheFz3 Regulate the Canonical Wnt Pathway and Direct Oral and Aboral-Specific Gene Expression (A) Activation of the canonical Wnt pathway in mid-blastula–stage control embryos (top row) and embryos derived from CheFz1-Mo (middle row)– or CheFz3-Mo (bottom row)–injected eggs, visualised by injecting eggs prior to fertilisation with RNA coding for a β-catenin–Venus fusion protein. Coinjected rhodamine dextran was distributed uniformly (not shown). β-catenin–Venus was stabilised specifically in the oral half of control embryos. CheFz1-Mo reduced β-catenin–Venus to barely detectable levels, while CheFz3-Mo caused it to accumulate in all cells. (B) Characteristic phenotypes of embryos derived from CheFz1-Mo– or CheFz3-Mo–injected eggs compared with uninjected controls. Differential interference contrast images of early gastrula-stage embryos: arrows indicate ingressing cells. CheFz1-Mo severely reduced the extent of cell ingression, although did not prevent oral thickening of the epidermal layer, while CheFz3-Mo caused expansion of the zone of cell ingression across most of the embryo. (C) Confocal images of similar early gastrula-stage embryos, with cell contours visualised using fluorescent phalloidin (green) and nuclei with ToPro3 (red). (D) Equivalent images of planula-stage embryos (1 d after fertilisation). There is a clear deficit in endoderm formation in embryos of CheFz1-Mo–injected but not in control CheFz1-5mp-Mo–injected embryos. CheFz3-Mo embryos show both endoderm and ectoderm, but oral–aboral polarity is disrupted. (E) Representative in situ hybridisation images of early gastrula embryos showing abolition of the oral CheBra expression territory following CheFz1-Mo injection, and expansion following CheFz3-Mo injection. (F) Aboral CheFoxQ2a expression at the same stage was abolished following CheFz3-Mo injection but expanded following CheFz3-Mo injection. In all panels, embryos are oriented with the oral pole up. Bars = 40 μm.

The oral localisation of maternal CheFz1 RNA placed it as a strong candidate for an oral/endoderm determinant acting upstream of β-catenin stabilisation. Functional tests using a morpholino antisense oligonucleotide targeted to the translation initiation site (CheFz1-Mo) supported this hypothesis (Figure 3). To assess Wnt pathway activation we injected eggs before fertilisation with RNA encoding a *Podocoryne* β-catenin–Venus fusion protein (see Materials and Methods). In controls, β-catenin–Venus was detected in a restricted territory covering approximately half of the embryo from the 16- to 32-cell stage onwards, with a sharp boundary developing by the mid-blastula stage (Figure 3A), as reported in *Nematostella* with *Xenopus* and *Nematostella* β-catenin–GFP fusion proteins [9]. Following CheFz1-Mo injection, β-catenin–Venus was barely detectable at the mid-blastula stage, indicating that active degradation of the protein, normally restricted to the aboral territory, had been promoted in all cells. As in the *Nematostella* study, we confirmed that the distribution of exogenous fluorescent protein faithfully reflected that of endogenous β-catenin by immunofluoresence of control cleavage-stage embryos with an anti–β-catenin antibody (unpublished data).

**Figure 4:**
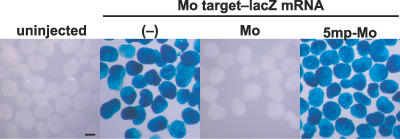
Specific Inhibition of Translation by CheFz1-Mo X-gal staining of embryos derived from eggs injected with a β-galactosidase reporter RNA bearing the CheFz1-Mo 5′ UTR target sequence. Co-injection of CheFz1-Mo but not CheFz1-5mp-Mo completely abolished β-galactosidase expression.

CheFz1-Mo–injected embryos showed severe reduction in the size of the oral territory of ingressing cells at the onset of gastrulation (Figure 3B and 3C), and consequently a large deficit in the amount of endoderm present at the end of gastrulation period (Figure 3D). Correspondingly, they showed strong down-regulation of the *Brachyury* gene *CheBra,* normally expressed prior to gastrulation in a small oral domain corresponding to the site of cell ingression [29] (E.H. and S. Chevalier, unpublished data; Figure 3E). CheFz1-Mo not only inhibited oral *CheBra* expression and gastrulation, but affected aboral patterning, as demonstrated by expansion of the aboral expression domain of *CheFoxQ2a* [24] (Figure 3F). A control morpholino containing five nucleotide substitutions (CheFz1-5mp-Mo) had no effect on development (Figure 3D and unpublished data). To confirm that CheFz1-Mo specifically blocked translation of the corresponding RNA target sequence, it was co-injected with a reporter RNA in which the CheFz1 5′ UTR target sequence was placed upstream of the β-galactosidase–coding sequence. CheFz1-Mo, but not CheFz1-5mp-Mo, completely abolished β-galactosidase expression (Figure 4).

These results indicate that the receptor CheFz1 acts as a classic positive regulator of the canonical Wnt pathway. Its localisation to the oral side of the early *Clytia* embryo is thus likely to be a key factor in the asymmetric activation of this pathway, and thereby in defining an oral gene expression territory from which endoderm derives [9,15].

In addition to the defects in gastrulation, CheFz1-Mo–injected embryos showed strikingly impaired swimming behaviour, turning slowly on themselves rather than developing directional movement during the early gastrula period. This undirected swimming phenotype led us to suspect that the CheFz1-Mo had interfered with the development of ectodermal polarity. Visualisation of cilia using an antiacetylated tubulin antibody supported this hypothesis; Cilia in CheFz1-Mo–injected embryos were short, curled-up, and lacked the oral–aboral alignment seen in control embryos (unpublished data). This phenotype is reminiscent of that described in vertebrate embryos following interference with a noncanonical Frizzled-mediated response known as planar cell polarity (PCP) [30]. Also consistent with interference in PCP, CheFz1-Mo–injected *Clytia* embryos failed to elongate, a process driven by intercalation of polarised epithelial cells [14]. In vertebrates, interference with PCP likewise disrupts the “convergent extension” movements responsible for embryo elongation during gastrulation [31,32]. These indications suggest that the receptor CheFz1 may be required for the development of PCP as well as for canonical Wnt signalling in *Clytia.*

It should be noted that CheFz1-Mo did not completely prevent either gastrulation or polarity development. Although the blastocoel remained mainly empty, some cell ingression was observed, and embryos adopted a pear shape rather than an elongated shape by the end of the normal gastrulation period (Figure 3D). This could be accounted for by incomplete inhibition of translation, by the presence of maternal CheFz1 protein, or to the involvement of independent pathways.

### CheFz3 Opposes CheFz1 Function

In line with its aboral localisation, CheFz3 function was found to oppose that of CheFz1, repressing canonical Wnt signalling and promoting aboral gene expression (Figure 3). Thus, embryos injected with the specific morpholino CheFz3-Mo showed β-catenin–Venus stabilisation across the entire embryo (Figure 3A). Correspondingly, cell ingression at gastrulation in CheFz3-Mo–injected embryos was initiated over a much increased area (70%–80% of the blastula surface compared with 20%–30% in uninjected siblings; Figure 3B–3D), the *CheBra* expression domain expanded (Figure 3E), and expression of *FoxQ2a* [24] was abolished (Figure 3F). We conclude that CheFz3 plays an important role in defining an aboral domain during early development.

The observation of global β-catenin stabilisation in CheFz3-Mo–injected embryos does not prove that CheFz3 functions directly to downregulate canonical Wnt signalling, because this effect could be due to β-catenin independent downregulation of CheFz1 RNA levels (see below). It should be noted, however, that the aboral localisation of CheFz3 in control embryos at the blastula and gastrula stages is much tighter than the graded oral localisation of CheFz1 (Figure 2), and matches more closely the sharply demarcated pattern of β-catenin stabilisation (Figure 3A), supporting the hypothesis of direct antagonism at the level of the Wnt pathway.

### Mutual Downregulation of *CheFz1* and *CheFz3*

**Figure 5:**
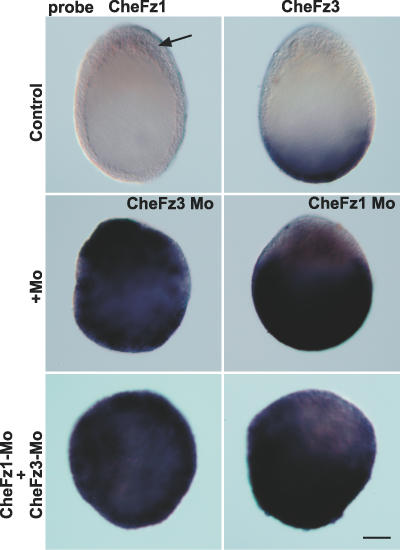
Reciprocal Regulation of CheFz1 and CheFz3 Representative in situ hybridisation images obtained with CheFz1 and CheFz3 probes on control and morpholino-injected embryos. The arrow points to the weak oral CheFz1 expression domain in controls at this stage. Each morpholino causes massive up-regulation of the other RNA, albeit with vestiges of an oral–aboral gradient still discernable. A mixture of both morpholinos caused simultaneous up-regulation of both RNAs.

Canonical Wnt pathway activation in many systems invokes a complex system of feedback regulation involving modulation of expression of multiple regulatory components to shape the β-catenin stabilisation domain [3]. In addition to Wnt pathway antagonism, the action of CheFz3 in the aboral domain of the early embryo was found to include strong negative regulation of *CheFz1.* A dramatic increase in the CheFz1 RNA level and a massive expansion of its territory was observed following CheFz3-Mo injection (Figure 5). Reciprocally, CheFz1-Mo promoted greatly increased CheFz3 RNA levels across the embryo, indicating mutual repression between the two genes at the level of new transcription or possibly RNA degradation. Despite the increased levels CheFz1 or CheFz3 RNA levels in these experiments, polarised distributions were still discernable. In particular, levels of CheFz3 RNA around the oral pole were always relatively low (Figure 5).

**Figure 6:**
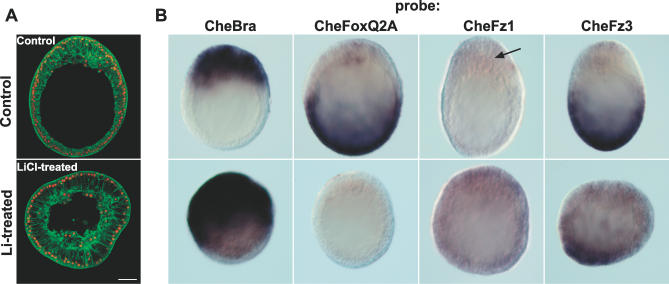
Global β-Catenin Stabilisation Uncouples Oralisation from CheFz RNA Levels (A) Confocal images of phalloidin/ToPro3-stained early gastrula embryos (see Figure 2) showing characteristic phenotypes obtained following Wnt pathway activation by treatment with 50 mM LiCl from the two-cell stage (bottom row) compared with untreated controls (top row). (B) Representative in situ hybridisation images of control and LiCl-treated embryos fixed at the early gastrula stage, with probes as indicated (weak oral CheFz1 expression indicated by arrows in controls).

A simple explanation for the mutual down-regulation between *CheFz1* and *CheFz3* would be that the level of both RNAs is regulated directly by canonical Wnt pathway activation, CheFz1 positively and CheFz3 negatively. Additional experiments showed that this is unlikely to be the sole explanation. Firstly, co-injection of the two morpholinos together resulted in simultaneous up regulation of both RNAs (Figure 5), a situation incompatible with opposing regulation by a single pathway. Secondly, treatment with LiCl (an inhibitor of the negative regulator GSK3β) to mimic canonical pathway activation [9] resulted in uncoupling of CheFz RNA levels from oral–aboral patterning. Like CheFz3-Mo injection, LiCl treatment caused strong oralisation of the embryo, as indicated by expansion of the gastrulation site (Figure 6A) and of the oral *CheBra* expression domain, and abolition of CheFoxQ2a expression (compare Figure 6B with Figure 3E and 3F). The effects of lithium on the levels of CheFz1 and CheFz3 RNAs were, however, much weaker than the dramatic effects of CheFzMo injection (compare Figure 6B with Figure 5). LiCl treatment was also unable to reverse the expansion of the CheFz3 RNA domain provoked by CheFz1-Mo (unpublished data). Taken together, these experiments indicate that β-catenin–independent mechanisms, for instance involving secondary secreted inhibitors, participate in the reciprocal downregulation between CheFz3 and CheFz1.

### Ectopic CheFz1 or CheFz3 Can Redirect Embryonic Polarity

**Figure 7:**
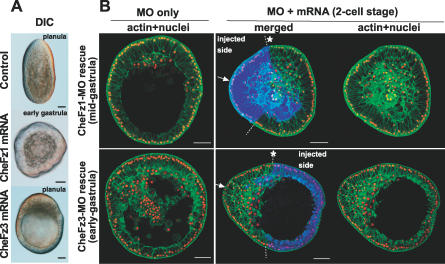
Embryo Polarity Redirected by Ectopic CheFz1 or CheFz3 (A) Differential interference contrast images of embryos following injection of 0.5 mg/ml RNA coding for CheFz1 or CheFz3 into eggs before fertilisation compared to uninjected controls. Stages as indicated. (B) Confocal images of early gastrula embryos stained with phalloidin/ToPro3 following morpholino injection into the egg (left column) and with polarity restored by injection of 0.5 mg/ml RNA (lacking the morpholino target site) into one blastomere at the two-cell stage (right two columns). Top row: CheFz1 misexpression; Bottom row: CheFz3 misexpression. Blue indicates the progeny of the RNA-injected cell revealed by fluorescent dextran. Arrows indicate ectopic pointed oral poles. The original position of the egg animal pole is deduced to lie on the injected–uninjected boundary (asterisk) since the first cleavage passes through the animal pole, and can be further pinpointed by residual endogenous polarity in CheFz1-injected embryos. Bars = 40 μm.

The morpholino loss-of-function experiments described above showed that CheFz1 and CheFz3 proteins are required for oral–aboral patterning in *Clytia.* To test whether these localised molecules were able to act as determinants to direct formation of the embryonic axis, we relocalised them by injecting synthetic RNA into one blastomere of two-cell stage embryos preinjected with morpholino. The effect of each synthetic RNA was first tested by injection into eggs before fertilisation. Injection of CheFz1RNA into eggs before fertilisation reproducibly caused an oralisation strongly reminiscent of the CheFz3-Mo phenotype, while CheFz3 RNA injection at moderate doses caused a gastrulation block as seen with CheFz1-Mo (Figure 7A). At high doses, CheFz3 RNA had an oralising effect resembling that of CheFz1RNA (not shown), suggesting that CheFz3 has a weak ability to activate the canonical Wnt pathway. A weak Wnt signalling ability has similarly been reported for the related protein *Drosophila* Fz3, which attenuates canonical Wnt pathway activation by Fz2 during wing development [33,34].

In accordance with a role as an oral fate determinant, CheFz1 RNA injected into one blastomere at the two-cell stage promoted a significant rescue of the CheFz1-Mo–induced gastrulation block. The restored endoderm was composed of descendents of both injected and uninjected blastomeres (Figure 7B), again indicating the involvement of secondary signalling mechanisms. Importantly, mislocalised CheFz1 caused formation of an ectopic pointed oral pole (arrowed in Figure 7B), centred among the progeny of the Fz1RNA-injected blastomere and distinct from the residual endogenous oral pole (asterisk in Figure 7B). Thus, localised CheFz1 is able to direct embryonic axis formation in *Clytia.*

Remarkably, we found that CheFz3 RNA could also redirect polarity development when introduced ectopically into CheFz3-Mo–injected embryos. In this case, ectopic oral poles became positioned opposite a domain of suppressed gastrulation corresponding to the progeny of the RNA-injected blastomere (Figure 7B). Ectopic oral poles were obtained in nine of 12 embryos in the CheFz1 rescue experiments and nine of 21 embryos in CheFz3 rescue experiments, the more variable results with CheFz3 reflecting greater sensitivity of injected blastomeres to RNA dose (see above).

Taken together, these RNA misexpression experiments indicate that an asymmetric distribution of either CheFrz1or CheFrz3 can direct polarisation of the *Clytia* embryo during pregastrula development.

## Discussion

**Figure 8:**
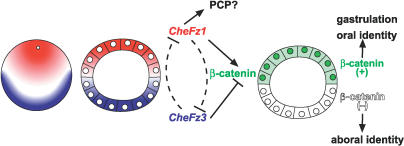
Model for Cnidarian Embryonic Patterning Initiated by Localised Maternal Frizzled RNAs CheFz1 (red) transcribed from animally localised RNA activates the canonical Wnt pathway to stabilise β-catenin (green) and so promotes development of oral fates (including gastrulation and endoderm formation). It may also play a role in the establishment of ectodermal polarity via PCP. CheFz3 (blue) transcribed from vegetally localised RNA spatially restricts canonical Wnt pathway activation by CheFz1, defining an aboral gene expression territory. The oral and aboral domains are reinforced by mutual downregulation of CheFz1 and CheFz3 (dotted lines), by mechanisms probably involving secondary signalling molecules.

Inheritance of localised determinants from different regions of the egg has long been known to provide an important mechanism for initiating embryonic patterning, and identification of determinants at the molecular level remains an important goal in developmental biology. We have identified an animally localised maternal determinant that directs oral–aboral axis development in the cnidarian *Clytia,* as the Frizzled-encoding RNA, CheFz1. In addition, we uncovered a second Frizzled RNA with an unanticipated vegetal/aboral localisation and an opposing function, which also acts as an axis determinant. The two Frizzled proteins set up a dynamic system for the regulation of canonical Wnt signalling along the oral–aboral axis, involving mutual downregulation by both direct and indirect mechanisms (see model in Figure 8). The Frizzled proteins may also have a second function, in directing global oral–aboral polarity via PCP operating in the ectoderm. Our study has a number of implications concerning the evolution of early embryonic patterning mechanisms in multicellular animals, notably that maternal determinant identity has not been evolutionarily maintained.

### Frizzled RNAs as Maternal Axis Determinants in *Clytia*

The Frizzled receptors CheFz1 and CheFz3 both fulfilled all the experimental requirements to qualify as maternal localised determinants. First, their RNAs were found to be localised in the egg (to the future oral and aboral poles), and to be inherited by oral- and aboral-fated territories, respectively. Second, loss-of-function experiments by morpholino-mediated translational inhibition showed that CheFz1 is required for oral specific gene expression and CheFz3 for aboral gene expression, and that the two proteins together define a domain of canonical Wnt signalling in the oral half of the embryo. The specificity of the morpholino experiments was confirmed by overexpression experiments, in which injection of CheFz3 RNA injection mimicked CheFz1-Mo and vice versa. Finally, mislocalised RNA of either CheFz1 or CheFz3 was able to restore and redirect axis development in morpholino-injected embryos.

Are CheFz1 and CheFz3 the sole localised determinants upstream of canonical Wnt signaling in the *Clytia* embryo? They are certainly dominant ones, since overexpression and misexpression experiments demonstrate that all other components necessary to support activation of this pathway are available throughout the embryo. Nevertheless, many Wnt pathway activators and inhibitors upstream or downstream of Frizzled could potentially adopt graded distributions at the RNA and/or protein levels and so contribute to axial patterning in undisturbed embryos, accounting for the residual polarity detected in CheFz1-Mo–, CheFz3-Mo–, and CheFz3 RNA–injected embryos. In *Nematostella,* none of the Wnt genes repertoired in the genome show detectable maternal RNA [18,23], although low maternal expression of one or more of them cannot be ruled out. The *Hydra* Wnt gene *HyWnt* likewise appears not to be expressed maternally [35]. Interestingly, however, a recent study in *Hydractinia* revealed that RNAs coding for the equivalent Wnt ligand, as well as for the downstream transcriptional activator Tcf, are present maternally and concentrated at the animal pole of the egg [19]. As with CheFz1 RNA, the asymmetric distribution of these two RNAs becomes accentuated as zygotic transcription occurs to produce a strong localisation at the future oral pole of the embryo, suggesting that as in *Clytia,* active feedback mechanisms are operating to reinforce polarity.

Cnidarian eggs may also contain other determinants acting in parallel to the Wnt pathway. In *Podocoryne,* maternal RNAs coding for Brachyury [29] and for the homeobox transcription factor Cnox4-Pc [36] are localised around the animal pole of the egg, and could potentially participate in directing gastrulation and/or other aspects of oral fate [37]. It will be instructive to test the maternal function of these molecules, and of the variety of Wnt pathway regulators present in cnidarians [18].

### Frizzled Receptors with Opposing Activities

The presence of an animally localised canonical Wnt pathway activator in *Clytia* was predicted from previous work (see Introduction); however, the involvement of a variant Frizzled protein acting to oppose Wnt pathway activation was unexpected. Our data suggest that the opposing function of CheFz3 involves both direct antagonism of CheFz1-mediated Wnt signalling and indirect mechanisms involving secondary molecules.

Direct antagonism of canonical Wnt pathway signalling by Frizzled family proteins has some precedent in other systems: *Drosophila* Fz3 will attenuate the response of Fz2 to Wnt ligand during wing development [33], while *Caenorhabditis* MOM5 appears to antagonise canonical Wnt signalling in the absence of ligand [38]. The antagonistic action of Fz3 and MOM5 is thought to be due to an alteration in the C terminal K-T-xxx-W motif implicated in Dishevelled binding [26]. CheFz3 is phylogenetically related to Dfz3 (Figure 1B) and appears similarly to have a weak ability to stimulate canonical Wnt signalling, but does not show an alteration in this motif. It will be interesting to determine the molecular basic of its antagonistic behaviour. Many possible sequence changes could potentially abrogate receptor function and render Frizzled molecules antagonistic, as for instance in the extracellular cysteine-rich domain of mouse Frizzled-1 [39]. It is also possible that noncanonical Wnt signalling through CheFz3 may antagonise canonical CheFz1 signalling, as has been reported for certain Wnt ligands [40,41].

Our data include several indications that indirect mechanisms contribute to the negative effect of CheFz3 on canonical Wnt signalling. Most striking was the dramatic effect of morpholino-mediated inhibition of either CheFz1 or CheFz3 RNA translation on RNA levels of the other, which extended well beyond the corresponding RNA domains and was at least partially LiCl insensitive. These observations could be explained by the involvement of downstream-secreted molecules that diffuse away from their sites of production in both oral and aboral territories and act as inhibitors of the opposing fates. Such mechanisms are characteristic of the dynamic regulation systems used frequently during embryonic development to maintain signalling activity gradients, for instance in the vertebrate organiser [42], and are predicted to provide the basis for the regulative properties typical of cnidarians [43]. Possible candidates as diffusible CheFz1 antagonists produced downstream of CheFz3 are the Dickkopf family proteins, shown to be expressed aborally in the *Nematostella* embryo and implicated in Wnt antagonism in *Hydra* polyps [18,44,45].

### Global Embryonic Polarity in Cnidarian Development

“Global” polarity is manifest in hydrozoan embryos and planula larvae by the common orientation of cilia within the ectoderm, responsible for their directed swimming. This global polarity confers certain remarkable properties, as revealed by bisection, grafting, and cell reassociation experiments, in which small pieces of blastula tissue can entrain the polarity of embryos reformed from disaggregated cells [17,37]. PCP, a noncanonical Frizzled-mediated process which acts to coordinate polarity of individual cells along a gradient of Frizzled activity [46], is highly likely to participate in global polarity in hydrozoans. It is becoming increasingly apparent that PCP plays an important role in the coordination of morphogenetic movements during animal development, notably in vertebrate convergent extension [31,32,47]. In *Clytia,* CheFz1-Mo injection caused disruption both of cilia alignment and of embryo elongation by convergent extension-like cell intercalation, phenotypes consistent with disruption of PCP. Although such effects could be indirect, and dependent on events downstream of canonical Wnt signalling, it is tempting to speculate that CheFz1 directly mediates both canonical Wnt signalling and PCP, these two pathways acting in parallel to direct regionalised gene expression and global polarity, respectively. This possibility is supported by experiments in *Podocoryne,* where development of global polarity can be uncoupled experimentally from specification of the endoderm territory at the oral pole [15]. It will be important to analyse the role for PCP in the development of global polarity in hydrozaon embryos by independent means, such as by monitoring the polarised intercellular localisation of Frizzled, Disheveled, Prickle, and Strabismus proteins [18,32].

### Evolution of Axial Patterning

On the basis of its use in cnidarians, regionalised canonical Wnt signalling has been proposed to have had an ancestral role in early embryo patterning in metazoans [9,11]. Its primary role appears to be in axial patterning, with Wnt pathway activation and expression of numerous regulators coinciding with the developing oral–aboral axis in diverse cnidarian species [18,19]. Ancestral axial patterning may also have involved members of a second group of signalling molecules heavily implicated in bilaterian embryos, the transforming frowth factor β (TGFβ) superfamily. A number of TGFβ ligands, antagonists, and downstream regulators have been found to show asymmetric expression patterns in embryos of both anthozoan and hydrozoan cnidarians [48–52]. Intriguingly, expression of many TGFβ pathway regulators is offset from the oral–aboral axis and may reveal a cryptic second embryonic axis, although roles in germ-layer and primary axis formations have also been suggested [49,50]. Functional studies are required to understand the significance of these expression patterns.

An important unresolved question concerns the relationship between axial patterning and germ layer specification. In *Clytia, Podocoryne,* and *Nematostella,* species which gastrulate by invagination and/or unipolar cell ingression from the oral pole [14,15,53], definition of the presumptive endoderm territory and of the oral pole are clearly closely linked, and both promoted by Wnt pathway activation. This is not the case in all cnidarians [37]. In *Hydractinia,* for example*,* endoderm forms in a nonpolarised fashion from internal cells of solid morula, and GSK3β inhibitor studies indicate that endoderm formation is uncoupled from Wnt pathway–specified axial patterning [19]. We suspect that in *Clytia,* endodermal fate is determined secondarily within the β-catenin/oral fate territory by a dynamic mechanism involving other signalling pathways, perhaps by TGFβ signalling as hypothesised in *Nematostella* [50]. Such an indirect role for the Wnt pathway in germ-layer specification would explain why experimental β-catenin stabilisation does not convert all cells to an endodermal fate [9,37; this study]. Vestiges of roles for the Wnt signalling pathway in axis specification (echinoderms, amphibians, and fish) and germ-layer specification (echinoderms, ascidians) can be detected in deuterostomes, but appear to have been largely superceded by other axis and germ-layer specification mechanisms in protostome models.

### Evolution of Wnt-Activating Determinants

The search for maternal determinants responsible for initiating regionalised canonical Wnt signalling in different animals is far from complete, but existing data suggest that evolutionary conservation does not extend to determinant identity. Apart from the *Clytia* Frizzled RNAs identified in this study, *Xenopus* Wnt11 RNA is the only clear example of such a localised determinant. Wnt11 RNA is localised to the vegetal cortex of the *Xenopus* oocyte, and both RNA and protein become concentrated on the dorsal side of the early embryo [54,55]. Maternal Wnt11 is necessary for formation of the dorsal organiser region and promotes dorsalisation [22]. It may, however, not be the sole localised activator of the Wnt pathway in *Xenopus:* localisation of Dishevelled, GSK3β binding protein (GBP), and even β-catenin itself may also contribute [56–58]. Likewise, Wnt and Tcf RNAs may be involved along with Frizzled RNAs in cnidarians [19; see above]. In zebrafish, maternal determinants that activate Wnt pathway signalling appear to act at the level, or upstream, of Frizzled [59,60], while in sea urchins, the Dishevelled protein adopts a localization appropriate for determinant activity, although other unidentified localised factors contribute to its activation [61]. On current evidence it thus appears that during metazoan evolution, a variety of Wnt pathway regulators have been adopted as maternal determinants. This situation may have been favoured by the existence of efficient systems involving positive and negative feedback mechanisms that provide rapid reinforcement of initially weak gradients of Wnt signalling activity, as demonstrated here in *Clytia.* Such mechanisms are likely to underlie the remarkable self-organising properties of cnidarians, and have been shown to allow Wnt activity “organising centres” to emerge from nearly uniform cell populations [43]. We can speculate that embryonic polarity specification in the bilaterian/cnidarian ancestor may have relied on consolidation by intercellular signalling systems of weak (possibly stochastic) asymmetries in the distribution of one or other Wnt pathway component in the egg or early embryo. As egg size, cleavage patterns, and other features of early development subsequently diversified, the availability of multiple potential cellular mechanisms for the localisation of Wnt pathway-activating proteins or their RNAs within the egg appears to have allowed a wide choice of determinant identity.

## Materials and Methods

### CheFz1 and CheFz3 cDNAs.

CheFz1 was isolated from a Triplex phage cDNA library following PCR using degenerate primers corresponding to conserved regions of metazoan Frizzled genes. The CheFz3 sequence was retrieved from an expressed sequence tag collection [24]. Phylogenetic relationships were determined by maximum likelihood analysis of aligned conserved Frizzled domains using PhyML software (http://atgc.lirmm.fr/phyml).

### Morpholinos.

Antisense morpholino oligonucleotides (GeneTools LLC, http://www.gene-tools.com) were designed to match the sequence of our clonal *Clytia* colonies (CheFz1-Mo: CCGTTCTTGTAAAAACAATTATGTC; CheFz3-Mo: ACGTTAGGCAGCATCACTGCTCCTT) and microinjected at 0.5 mM prior to fertilisation. A mismatch morpholino CheFz1-5mp-Mo (CCGTTGTTCTAATAAGAATTATCTG) was used to confirm specificity.

To confirm the effects of morpholinos on translation, mRNA encoding β-galactosidase downstream of CheFz1 5′ UTR (positions −63 to −1 of the start ATG) was synthesised and injected into eggs with or without morpholino oligonucleotides before fertilisation. Resultant embryos were fixed and stained with X-gal substrate [62].

### Embryo manipulation and microinjection.

Gametes were obtained from C. hemisphaerica adult medusae generated from established laboratory colonies as described previously [24]. Embryos were raised in Millipore filtered seawater at 16–18 °C. Microinjection was performed with an Eppendorf “Femtojet” (injection volumes estimated at 3% of egg or blastomere volume; http://www.eppendorf.com). RNAs for microinjection were transcribed from the CheFz1 and CheFz3 cDNA open reading frames using T3 mMessage machine (Ambion, http://www.ambion.com) following subcloning into the pRN3 plasmid [63].

In situ hybridisation using DIG-labelled antisense RNA probes was performed as described previously from cDNAs corresponding to CheFoxQ2a [24]; CheBra (cloned from early embryo cDNA: S. Chevalier and E.H., unpublished data), CheFz1, and CheFz3.

### Microscopy and β-catenin–Venus visualisation.

To visualise cell boundaries and nuclei, embryos were fixed using 4% formaldehyde and stained with 130 nM fluorescent phalloidin and 0.4 μM ToPro3 (both from Molecular Probes, http://probes.invitrogen.com), respectively.

To monitor activation of the canonical Wnt pathway, eggs were injected prior to fertilisation with 0.5 mg/ml RNA coding for full-length Podocoryne carne β-catenin fused to the Venus GFP variant [64]. This was transcribed from a construction in pRN3, with the Venus sequence placed at the C-terminal end of β-catenin, following a three-codon linker. Embryos were imaged live or after fixation in methanol at −20 °C. Confocal microscopy was performed using a Leica SP2 microscope (http://www.leica.com).

## Supporting Information

### Accession Numbers

The GenBank (http://www.ncbi.nim.nih.gov/Genbank) accession numbers for the structures discussed in this paper are CheFz1 (DQ869571), CheFz3 (DQ869572), CheBra (DQ872898), and β-catenin *(P. carne)* (DQ869570). *N. vectensis frizzled* gene sequences were recovered by BLAST from the Joint Genome Institute N. vectensis genome project trace files.

## Abbreviations

PCP: planar cell polarity
Mo: morpholino
TGFβ: transforming growth factor β
